# Irreversible Electroporation: An Emerging Immunomodulatory Therapy on Solid Tumors

**DOI:** 10.3389/fimmu.2021.811726

**Published:** 2022-01-07

**Authors:** Nana Zhang, Zhuoqun Li, Xuan Han, Ziyu Zhu, Zhujun Li, Yan Zhao, Zhijun Liu, Yi Lv

**Affiliations:** ^1^ Institute of Regenerative and Reconstructive Medicine, Med-X Institute, First Affiliated Hospital of Xi’an Jiaotong University, Xi’an, China; ^2^ National Local Joint Engineering Research Center for Precision Surgery & Regenerative Medicine, First Affiliated Hospital of Xi’an Jiaotong University, Xi’an, China; ^3^ Shaanxi Provincial Center for Regenerative Medicine and Surgical Engineering, First Affiliated Hospital of Xi’an Jiaotong University, Xi’an, China; ^4^ Department of Hepatobiliary Surgery, First Affiliated Hospital of Xi’an Jiaotong University, Xi’an, China

**Keywords:** irreversible electroporation, *in-situ* tumor vaccine, immune response, immunotherapy, tumor antigens, combination therapy

## Abstract

Irreversible electroporation (IRE), a novel non-thermal ablation technique, is utilized to ablate unresectable solid tumors and demonstrates favorable safety and efficacy in the clinic. IRE applies electric pulses to alter the cell transmembrane voltage and causes nanometer-sized membrane defects or pores in the cells, which leads to loss of cell homeostasis and ultimately results in cell death. The major drawbacks of IRE are incomplete ablation and susceptibility to recurrence, which limit its clinical application. Recent studies have shown that IRE promotes the massive release of intracellular concealed tumor antigens that become an “*in-situ* tumor vaccine,” inducing a potential antitumor immune response to kill residual tumor cells after ablation and inhibiting local recurrence and distant metastasis. Therefore, IRE can be regarded as a potential immunomodulatory therapy, and combined with immunotherapy, it can exhibit synergistic treatment effects on malignant tumors, which provides broad application prospects for tumor treatment. This work reviewed the current status of the clinical efficacy of IRE in tumor treatment, summarized the characteristics of local and systemic immune responses induced by IRE in tumor-bearing organisms, and analyzed the specific mechanisms of the IRE-induced immune response. Moreover, we reviewed the current research progress of IRE combined with immunotherapy in the treatment of solid tumors. Based on the findings, we present deficiencies of current preclinical studies of animal models and analyze possible reasons and solutions. We also propose possible demands for clinical research. This review aimed to provide theoretical and practical guidance for the combination of IRE with immunotherapy in the treatment of malignant tumors.

## Introduction

Irreversible electroporation (IRE), a novel physical ablation technique, applies a high-voltage pulsed electric field (PEF) to alter the cell transmembrane voltage, causes nanometer-sized membrane defects or pores in the cell, and eventually leads to loss of homeostasis and cell death (1, 2). It has been used to treat arrhythmic diseases and inactivate microorganisms (3–5). In 2005, Davalos et al. first applied IRE to destroy cancer cells (6). Recently, IRE has been widely utilized to ablate unresectable solid tumors in the clinic with favorable safety and efficacy (7–9).

As opposed to thermal ablative techniques, IRE induces cell death *via* the delivery of high-voltage short electrical pulses (EPs) and possesses several advantages as a non-thermal ablation technique: a) less collateral thermal damage, especially for vital nerves, vessels, and cavity structures; b) no heat sink effect, avoiding incomplete ablation due to the energy reduction caused by blood flow; and c) preservation of the extracellular matrix (ECM) scaffold, promoting rapid postoperative recovery (10, 11). Currently, IRE is performed mostly for solid tumors such as liver cancer (12), pancreatic cancer (13), and prostate cancer (8) and provides an advantageous palliative treatment for advanced tumors in the vicinity of important ductal structures, such as large blood vessels, the intestines, bile ducts, or the urinary tract. However, the uneven distribution of the PEF, resulting from the heterogeneous electrical properties of tumor tissue (14, 15), leads to incomplete ablation and increases the risk of tumor recurrence, which limits the popularity of IRE in clinical practice. Notably, the membrane perforation resulting from IRE can promote the massive release of intracellular concealed tumor antigens, inducing a potential antitumor immune response to kill residual tumor cells after ablation and inhibit the local recurrence of tumors (16).

Recent studies have shown that IRE also induces an excellent effect on activating local and systematic immune responses (9, 17). Therefore, IRE can be regarded as a potential immunomodulatory therapy.

Cancer treatment has entered the era of clinical multidisciplinary comprehensive treatment, and the prognosis of patients with a variety of tumors has been significantly improved (18). Recently, the immune response induced by IRE has gained much interest among researchers (19, 20). Several clinical studies have confirmed that IRE can induce a significant immune response in cancer patients and significantly improve antitumor efficacy (21–23). Therefore, IRE combined with immunotherapy might have synergistic effects on malignant tumor treatment. This review aimed to elucidate the characteristics and mechanisms of the IRE-induced immune response and its potential in combination with immunotherapy for the treatment of tumors. This study will provide theoretical and practical guidance for the clinical application of IRE combined with immunotherapy in the treatment of solid tumors.

## Effect of IRE on Tumors

### The Development of IRE as a Tumor Therapy

Electroporation, an old technique, applies an external PEF to increase cell membrane permeability, inducing the development of nanoscale metastable structure defects or “pores”, which are considered to be the source of enhanced permeability (24). The application of electroporation in the biomedical field began in 1982, and Neumann et al. first applied this strategy to introduce exogenous DNA into cells (25). During electrotransfection, the pores in the cell membrane may persist for a few seconds to a few minutes. After the entry of exogenous substances, cell membrane integrity can be restored without affecting cell survival, which is known as reversible electroporation (RE) (26). Later, many studies reported the important role of RE in introducing foreign molecules into living cells, and this strategy has been used for molecular and gene transfer *in vitro* for many years (27, 28).

**Figure 1:**
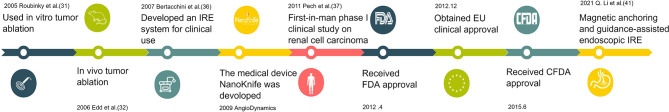
A timeline for the development of irreversible electroporation (IRE) on tumor treatment.

Electroporation technology for cancer therapy, known as electrochemotherapy (ECT), begins with chemotherapeutic drug delivery and promotes the absorption of drug molecules by tumor cells (29). However, when electric field strength (EFS) is higher than a certain threshold, excessive leakage of intracellular substances or slow closure of the cell membrane will cause irreversible damage to cells. Cell membrane surface perforation cannot be repaired, eventually leading to cell death (30). In 2005, Davalos et al. first announced that IRE can be used as a single therapy (without combined cytotoxic drugs or thermal effects) to destroy cancer tissue and named it “irreversible electroporation”. They found that IRE alone showed an excellent ability to destroy undesirable tissues in a similar manner to traditional focal thermal therapies (31). Edd et al. first used IRE *in vivo* for liver ablation in Sprague–Dawley rats in 2006 (32). Since then, a growing number of animal trials have investigated the safety and efficacy of IRE for oncology treatment (33–35). In 2007, Bertacchini et al. developed an irreversible electroporator approved for clinical use (36). This technology, marketed as NanoKnife, the first IRE tumor therapeutic apparatus, was developed by AngioDynamics in 2009. Pech et al. reported a first-in-human phase I clinical study of IRE in renal cell carcinoma in 2011 (37). Later, NanoKnife was approved by the FDA for clinical trials in April 2012. The tool was clinically licensed in the European Union in December of the same year, with CFDA approval to enter the Chinese market for clinical trials in June 2015, and is being used clinically for selected patients with locally advanced pancreatic cancer (38–40). Currently, researchers are devoted to developing therapeutic devices with customized IRE catheter-based electrodes delivered under endoscopy for surgical trauma reduction (41, 42). **Figure 1** shows the timeline for the development of IRE for tumor treatment. As the precise digital control system of IRE ensures accurate output parameters for EP (43) and various electrodes have been designed based on tumors in different organs, and even based on tumor morphology (44, 45), the ablation effect of IRE on tumors is more accurate and controllable than that of other ablation therapies (46, 47).

### Clinical Application of IRE on Tumors

The clinical trials of IRE ablation on tumors were often performed under computed tomography or ultrasound guidance using the commercially available NanoKnife system, which has three lengths of electrodes that are inserted into the tumor to achieve ablation (48). The number of electrodes used in the treatment procedure is determined by the size of the tumor. For lesions smaller than 2 cm, three electrodes are placed at the periphery of the lesion, and four electrodes are always placed at the periphery of the lesion for lesions between 2 and 3 cm. Four to six electrodes are used, including one to two electrodes in the center of the lesion, for lesions which are larger than 3 cm (7, 49, 50). However, a maximum of six electrodes can be used in the treatment process, as it is the maximum number of electrodes allowed by the IRE generator (7, 50). Compared with the electrodes used in most thermal ablation techniques, the electrodes used in IRE are relatively thin and allow complete destruction of the tumor and healthy liver tissue within a safe range of 1 cm around the tumor (49), and the optimal distance between the two electrodes is 0.7–2.0 cm (51). The maximum voltage and current that the generator can provide are 3,000 V and 50 A (52). Almost all clinical trials adopt an EFS of 1,500–1,800 V/cm and current of 20–50 A as the treatment parameters (52, 53). The pulse duration chosen for clinical trials is 70–100 μs, most commonly 90 or 100 μs (54). Usually, the efficacy is judged after a treatment of 90 pulses (50, 53, 55). If an insufficient extent of the ablation zone was suspected, IRE probes were repositioned, and another pulse application was performed (52). One study on pancreatic adenocarcinoma adjusted the pulse number from 90 pulses in one cycle to 30 pulses in three cycles because the patients developed interstitial edematous pancreatitis after IRE-1d (56). Technical success was defined as the ability to deliver the complete set of electrical pulses as planned (49). The above treatment parameters are not significantly different between various tumor types. Additionally, almost all clinical trials were performed percutaneously, and only some trials (54, 56, 57) used an open surgical route in a subset of cases, possibly related to the operability of the operation.

### Clinical Efficacy of IRE on Tumors

Recently, many clinical trials have evaluated the therapeutic effect of IRE as tumor therapy (**Supplementary Table 1**). Twelve studies (7, 48–55, 58–61) involving 295 cases explored the efficacy and safety of IRE on primary or secondary liver tumors. Some of the results indicated that the effectivity rate of IRE therapy ranged from 74% to 100% (49, 54, 55, 61). The main complications were bleeding, gastric ulceration, liver abscess, and myocardial infarction, and the highest incidence of complications was 40%. The average recurrence rate in these studies was higher than 20%. Only one study (62) reported no recurrence during the follow-up period of 7 months. A pilot study (63) demonstrated that IRE is a viable treatment option for hilar cholangiocarcinoma. Regarding the effect of IRE on locally advanced pancreatic cancer (LAPC), 13 studies (38, 56, 57, 62, 64–72) involving 391 patients have shown that the effectivity rate is more than 80% and even 100% (56, 62, 64, 66, 70) in some documents. The most attainable goal of IRE in the management of LAPC is the obvious palliation of symptoms. Investigators have carefully weighed the survival benefit and treatment-related complications (50, 54, 56, 67). Complications occurred in the studies, including upper gastrointestinal bleeding (38, 65, 67, 70, 73), bleeding duodenal ulcers (68), pancreatic fistulas (70), acute pancreatitis (57, 64, 67, 69), internal fistulas in the duodenum (66), and portal vein thrombosis (70), with incidence rates ranging from 10% to 40%. However, the recurrence rate was documented to be as high as 58% with a follow-up time of 18 months (65). Additionally, IRE is also increasingly being noted by researchers as a treatment for small-cell renal cancer. Two studies involving 22 patients reported effectivity rates of 57.1% and 93.3% (74, 75). One study on prostate cancer showed comparable efficacy of IRE to standard radical prostatectomy in terms of 5-year recurrence rate, and IRE showed better preservation of urogenital function (76). Two studies found good therapeutic value of IRE in metastatic cancer, including bilateral lung metastasis of osteosarcoma (77) and retroperitoneal metastasis of ovarian, gastric, and pancreatic cancer (78). Another study confirmed that IRE possesses an acceptable safety profile and is effective in eradicating difficult-to-reach colorectal liver metastases (CRLMs) (61), while another study showed that IRE is not effective for the treatment of lung malignancies (59).

Among the clinical trials, most studies considered CT or MRI as the primary modality for assessing IRE efficacy and possible complications. The complication incidence and tumor recurrence rates were associated with the duration of follow-up and the situation of the patients, but the effectiveness and safety of IRE in the treatment of hepatocellular carcinoma (HCC) and LAPC have been verified in extensive clinical trials. However, the high recurrence rate requires caution, as recurrent tumors always exhibit more aggressive behaviors (79). Therefore, combining IRE with other therapies may be an important strategy for achieving extended survival benefits.

## Characteristics of the Immune Response in Tumors Induced by IRE

### IRE Induces a Much Stronger Immune Response Than Other Ablation Therapies

The biological effects of IRE are based on the microsecond PEF. The microsecond EP action is extremely short, and rare heat is generated during this process, which can be diffused and absorbed rapidly (80). Therefore, IRE, independent of thermal effects, is a non-thermal ablation technique that only acts on the lipid bilayer of the cell membrane and has little effect on other molecules, such as membrane proteins and intracellular macromolecules (2, 11). In contrast, cryoablation and thermal ablation lead to protein denaturation, resulting in changes in tumor antigenicity (81). One study collected and analyzed cell lysates of B16 melanoma cells after exposure to heat (50°C, 30 min), cold (−80°C, 30 min), and IRE (1,250 V/cm, 99 pulses, 50 ms pulses, 1 Hz interval). The researchers found that IRE released the most protein and tyrosinase-related protein-2 antigen (TRP-2). IRE dramatically outperformed both cold and heat in T-cell activation (82). Another clinical study found that the levels of macrophage migration inhibitory factor (MIF) in the serum of liver patients increased significantly after IRE treatment compared with those in patients in the radiofrequency ablation (RFA) group. The axial diameter and area of the tumor ablation zone of the IRE group were significantly smaller than those of the RFA group after 1 year. This result was attributed to the immediate increase in IRE-mediated release of MIF, which promoted early tissue repair and shrinkage of the ablation zone (83). Bulvik et al. ablated normal liver tissues *via* RFA or IRE and found that RFA-treated liver tissues formed a clear inflammatory margin around the ablated area, whereas in IRE-ablated tissues, inflammatory cells could infiltrate and penetrate into the entire ablated area, with immune cells distributed along the residual blood vessels. The level of secretion of IL-6 in the IRE group was 3.3 times higher than that in the RFA group. The researchers further investigated the ablation effects of IRE and RFA in a mouse model of hepatocellular carcinoma. The results showed that IRE was more effective than RFA in ablating localized liver tumor tissue, and it also inhibited subcutaneously transplanted tumors in distant sites. The density of infiltrating immune cells was positively correlated with serum IL-6 levels (84). A subcutaneous pancreatic cancer mouse model study found that the number of infiltrating CD3^+^ T cells was significantly higher in the ablated tumor tissue at 6, 12, and 24 h in the IRE group than in the cryoablation group, and there were many more infiltrating macrophages in the IRE-ablated tumor tissue at 12 and 24 h than in the cryoablated tumor tissue (85). These results suggested that IRE can arouse a more robust immune response than other ablative therapies, and this focal ablated therapy can be designed to prime the immune system to function in concert with immunotherapies to eventually achieve improved and durable cancer treatment *in vivo*.

### The Local Immune Responses in Tumors Induced by IRE

**Table 1** summarizes the characteristics of local and systematic immune responses induced by IRE in preclinical and clinical studies in different solid tumors. Exploration of the change in the local tumor immune microenvironment (TIME) requires *in-situ* tumor tissue, the obtainment of which may result in new trauma to patients, and studies analyzing the local TIME have mostly used animal models. Several studies (86–89) of liver tumor model rats and mice (86–89) and dogs (90) showed an increased density of localized CD3^+^ T cells (90), CD8^+^ T cells (86, 87, 89), dendritic cells (DCs) (86), and macrophages (88) and a decreased number of Treg cells (86, 87) and PD-1^+^ T cells (86) in the tumor tissue after IRE treatment. Two studies of IRE ablation of pancreatic cancer in mice (85, 91) indicated increased infiltration of CD3^+^ T cells (85), CD45^+^ T cells (91), CD8^+^ T cells (91), and macrophages (85) and higher levels of IFN-γ (91), CCL1 (91), and IL-2 (91) in local residual tumors of the IRE group. In contrast, the levels of cells such as myeloid-derived suppressor cells (MDSCs) (91) and cytokines such as IL-4 (91) and IL-6 (91) with tumor-promoting effects were decreased in the IRE-treated group.

**Table 1:** The characteristics of local and systematic immune responses induced by IRE in preclinical and clinical study of different tumor.

Species and tumor model	Detecting time	Detecting method	Effect of IRE on the immune status on tumor-bearing organism			
			Local Systematic			
			Tumor tissue	Spleen	Lymph nodes	peripheral blood
Murine subcutaneous liver cancer	IRE-7d	IHC, FCM	CD8+T, DC↑, Treg, PD-1+T↓	CD8+IFN-γ+T↑,Treg,PD-1+ T↓(86)	NA	NA
Murine orthotopic HCC	IRE-3d, 7d, 14d	FCM, CBA	CD8+T↑, Treg↓	NA	NA	IFN-γ, IL-2, TNF-α and IL1β↑, IL-10↓ (87)
Human HCC	IRE-1d, 3d, 7d	FCM	NA	NA	NA	Activated T cells, neutrophils, monocytes and NK ↑, Treg lymphocytes, CD4+T↓ (87)
Murine orthotopic HCC	IRE-2h, 2d, 7d, 14d, 90d	IHC, ELISA	Macrophages↑	NA	NA	IL-3, IL-13, IL-15, IL17A, IL-22, IL-28, IL-31 and IFN-γ↑ (88)
Murine orthotopic HCC	IRE-2h	IHC	CD8+T↑	NA	NA	CD8+T↑ (89)
Canin orthotopic HCC	IRE-4d	IHC	CD3+T↑ (90)	NA	NA	NA
Murine orthotopic PC	IRE-7d	IHC, FCM	CD45+T,CD8+T, IFN-γ,CCL1,IL-2↑; MDSCs,IL-4, IL-6↓(91)	NA	NA	NA
Human PC	Pre-IRE, IRE-2w, IRE-3m	NA	NA	NA	NA	Activated PD-1+T↑, Treg↓(92)
Human PC	Pre-IRE, IRE-1d, 3d, 5d	NA	NA	NA	NA	Treg↓ (93)
Human PC	Pre-IRE, IRE1d	FC	NA	NA	NA	CD4+CD25+T, CD4+CD25+FoxP3+T↓ ((56)
Rabbit cervical tumors	IRE-1d,1w,2w,3w	ELISA	NA	NA	NA	IL-1↑, IL-6↓ (94)
Murine orthotopic PC	IRE-0h,6h,12h and 24h	IHC	Macrophages, CD3+T↑ (85)	NA	NA	NA
Murine subcutaneous and orthotopic PC	IRE-7d	FC	CD8+T, memory CD4+T↑	CD8+T↑	Memory&effector CD8+T, Memory CD4+T↑ (95)	NA
Rat subcutaneous osteosarcoma	IRE-1d, 3d, 7d,14d, 21d	FCM, ELISA	NA	IFN-γ+T↑	NA	CD3+T, CD4+T and CD4+/CD8+T↑, IL-2R↓ (96)

*CBA: Cytometric bead array; ELISA: enzyme linked immunosorbent assay; FCM: flow cytometry; IRE: irreversible electroporation; IHC: immunohistochemistry; HCC: hepatocellular carcinoma; NA: not available; PC: pancreatic cancer; ↑: the cells or cytokines increased after IRE

↓: the cells or cytokines decreased after IRE.

### Systematic Immune Responses in Tumors Induced by IRE

We summarized the changes in immune cells and cytokines in the peripheral blood, lymph nodes, and spleen of tumor-bearing organisms after IRE treatment to analyze the characteristics of the IRE-mediated systemic immune response. There have been five studies (86–90) involving mouse and rat liver cancer models (86–89) and a dog liver cancer model (90) and one clinical study on liver cancer patients (87). The results showed an increased frequency of CD8^+^IFN-γ^+^ T cells (86) and a decreased number of Treg and PD-1^+^ T cells (86) in spleens in the IRE group. There was a higher proportion of CD8^+^ T cells (89), IFN-γ^+^ T cells (87, 88), neutrophils, monocytes, and NK cells (87) as well as higher levels of IL-2 (87), IL-3 (88), IL-13 (88), IL-15 (88), IL-17a (88), IL-22 (88), IL-28 (88), IL-31 (88), TNF-α (87), and IL-1β (87) and lower concentrations of CD4^+^ T cells (87), Treg cells (87), and IL-10 (87) in the peripheral blood of the IRE-treated group than in the control group.

Three murine pancreatic cancer model studies and three clinical studies of pancreatic cancer patients found that activated PD-1^+^ T cells (92) were increased and Treg cells (92, 93) were decreased in the peripheral blood. Moreover, there was an increased number of memory CD4^+^ T and CD8^+^ T cells as well as effector CD8^+^ T cells in the lymph nodes (94) after IRE treatment. A study of 20 rabbits with cervical cancer (95) found increased levels of IL-1 and IL-6 in peripheral blood after IRE ablation of tumors. Another study of osteosarcoma in rats (96) showed increased IFN-γ^+^ cells in the spleen and increased CD3^+^ T, CD4^+^ T, and CD4^+^/CD8^+^ T cells as well as decreased levels of IL-2R in the peripheral blood.

These results acknowledged that IRE induces obviously cellular immune responses in the local TIME as well as in systemic immune organs in tumor-bearing organisms. Most of the results suggested that IRE enhanced the density of immune cells and cytokines with antitumor effects while reducing the level of these cytokines with tumor-promoting effects. From the view of cancer-immune phenotypes (97), IRE can induce an immune-inflamed phenotype in the local tumor microenvironment (TME), and this profile suggests a clinical response to anti-PD-L1/PD-1 therapy (98, 99).

### IRE Induces an “*In-Situ* Tumor Vaccine” Effect

Ablative techniques induce antitumor immune responses by increasing the availability of tumor-specific antigens in an inflammatory context (100, 101). The specific antigens released from tumor cells are processed and presented by antigen-presenting cells, which enhance or induce an antitumor T-cell response (102). IRE was reported to significantly improve antitumor efficacy in immunocompetent mice but not in immunodeficient mice (91, 103). The immunocompetent tumor-bearing mice were rechallenged with the same cell line after 18 days of IRE treatment, and the growth of the second tumors was shown to be significantly reduced or entirely prevented. There was robust CD3^+^ cell infiltration in some treated mice, with immunocytes focused at the transition between viable and dead tumor cells. However, none of this was observed in immunodeficient mice (103). A hepatocellular carcinoma animal study showed that IRE-treated mice were tumor free after secondary tumor injection and showed increased splenic CD8^+^IFN-γ^+^ T cells. Depletion of CD8^+^ T cells induces local tumor regrowth and distant metastasis after IRE. In addition, inoculation of IRE-processed H22 (hepatocellular carcinoma cell line) cell lysates also prevented tumorigenesis in mice (86). Another study of orthotopic pancreatic cancer models found that an abscopal antitumor effect can be achieved after *in-situ* tumor ablation with IRE or exposure to the tumor culture supernatant of IRE-treated Panc02 cells (pancreatic cancer cell line) (95). The same animal model study showed that IRE can act as an “*in-situ* vaccine,” generating neoantigen-specific T cells that confer protection against tumor growth by adoptive cell transfer into treatment-naive immunocompromised mice (91). These results suggest that IRE can enhance tumor immunogenicity and increase the recognition of tumor antigens by the immune system to serve as a tumor vaccine.

## Mechanisms Related to the IRE-Induced Immune Response In Tumors

### IRE Mediates the Release of Damage-Related Molecular Patterns

**Figure 2:**
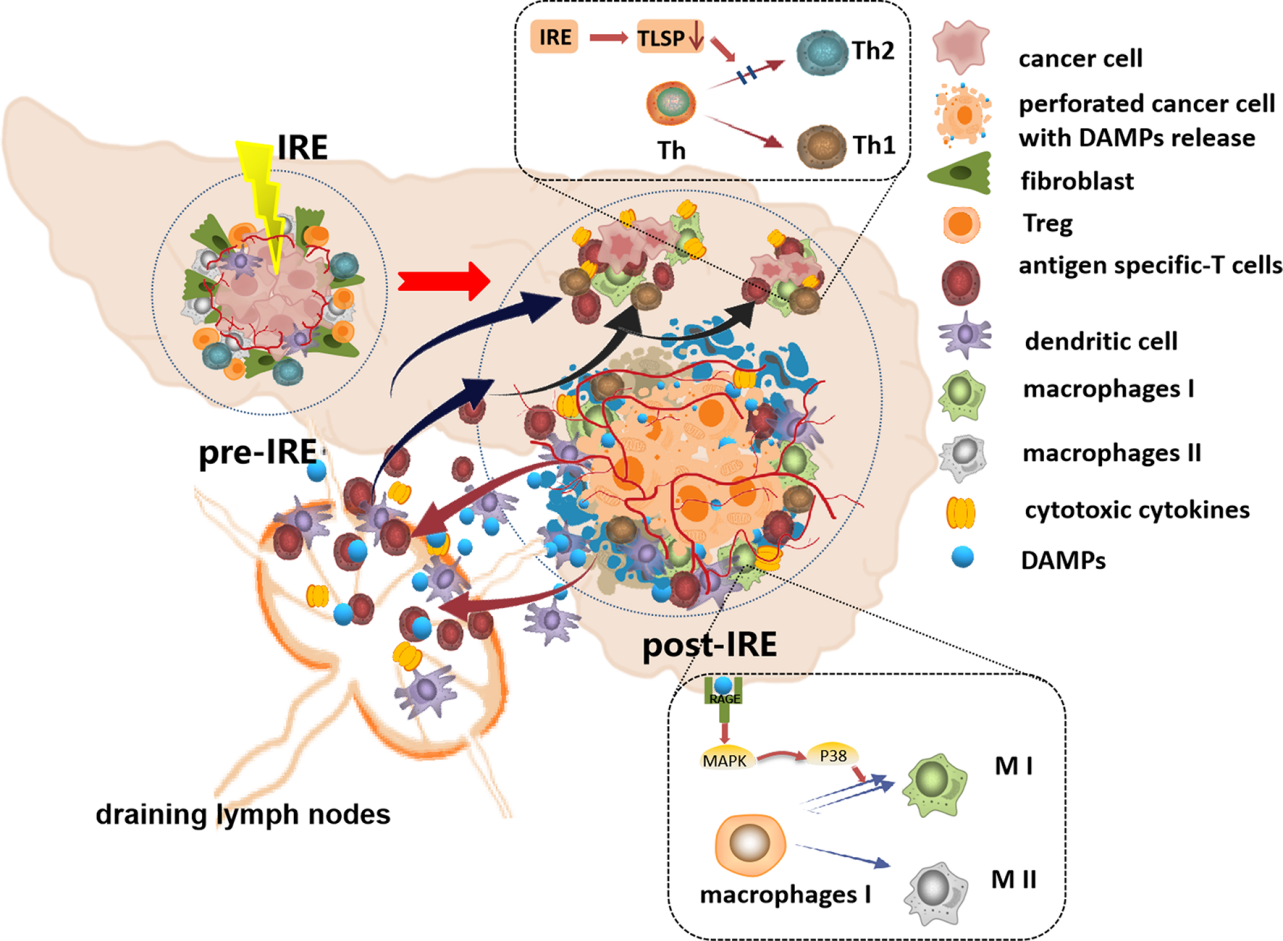
The schematic diagram of the reported mechanisms involved in IRE-induced immune responses. IRE increases the synthesis and secretion of damage-associated molecular patterns (DAMPs), and the DC cells take up these DAMPs, migrate to draining lymph nodes, and then activate tumor antigen-specific T cells, and the activated T cells home to tumor sites to eliminate the residual tumor cells. The DAMPs activated the MAPK–p38 pathway by binding to RAGE, resulting in M1 macrophage polarization. IRE inhibited TSLP in the TME preventing Th2 polarization. Additionally, IRE softens the ECM, increases the density and permeability of tumor vessels, and facilitates the infiltration of immune cells into residual tumor tissues.

Studies have proven that IRE can increase the synthesis and secretion of endogenous danger signaling molecules, namely, damage-associated molecular patterns (DAMPs), from damaged cells. DAMPs include ATP, high-mobility group B1 (HMGB1), calreticulin, and heat shock proteins (HSPs) and induce immunogenic cell death (ICD) (95, 104, 105). DCs resident in tumor tissues can take up these DAMPs and migrate to draining lymph nodes, activate tumor antigen-specific T cells, and affect the expansion of immunosuppressive T cells. Activated T cells home to remote sites to eliminate metastases and inhibit tumor progression (106–108). A study verified that the release of DAMPs increases with increasing EFS, and the DAMP secretion level is positively correlated with cell death (105). He et al. found that IRE increased the release of HMGB1, which activated the MAPK–p38 (mitogen activated protein kinases–p38) pathway by binding to the receptor for advanced glycation end products (RAGE), resulting in M1 macrophage polarization. In addition, M1 macrophage polarization was enhanced by the positive feedback-induced release or expression of HMGB1 and RAGE through the MAPK–ERK (MAPK–extracellular signal-regulated kinase) pathway in macrophages (104). The researchers also found that IRE-induced synthesis and secretion of HMGB1 promote specific T-cell infiltration and enhance immune memory. IRE not only led to complete tumor regression *in situ* but also induced an abscopal effect, suppressing the growth of latent lesions (95). Thymic stromal lymphopoietin (TSLP) has been demonstrated to drive immune cell polarization into the cancer-promoting Th2 immunophenotype in a variety of tumors (109, 110). Goswami et al. found that IRE inhibited the expression of TSLP in the TME of breast cancer in mice and humans and prevented immunosuppressive evolution of the TME (111). **Figure 2** depicts the reported mechanisms involved in IRE-induced immune responses.

### IRE Remodels the Tumor Microenvironment

As IRE is non-thermal, it maintains important ECM structures and preserves the complete structure of blood vessels in the tumor tissue, which can prompt the infiltration of subsequently primed effector T cells to the residual ablated tumor site (30, 84). A recent study found that microvessel density and permeability increased in the viable margin of tumor tissue after IRE. The microvessel density gradually decreased after 6 days of IRE ablation and returned to the baseline level on IRE-9d. In addition, the percentages of cells expressing the hypoxia markers hypoxia-inducible factor 1-α (HIF1-α) and carbonic anhydrase-9 (CA-IX) in the residual tumor area after IRE-4d were 53% and 24%, respectively, compared with the control group, respectively. The percentages of cells expressing hyaluronate-binding protein-1 (HABP1) (a marker of stromal hyaluronic acid) and lysine oxidase (LOX) (a marker of extracellular matrix stiffness) were 70% and 41%, respectively, compared with the control group. These proteins gradually increased after IRE-6d (108), suggesting that IRE can transiently improve the TME by increasing the density and permeability of tumor vessels, softening the ECM, and relieving hypoxia, which facilitated the infiltration of immune cells into residual tumor tissues (112, 113).

One study found that IRE triggered reactive oxygen species (ROS)-dependent apoptosis in pancreatic cancer cells mediated by inhibition of the PI3K–Akt pathway. This efficacy was synergistically enhanced by IRE combined with M1 virus administration (114). Another study on liver cancer showed that nsPEF induced the translocation and release of PD-L1 from the hepatoma cell membrane and promoted CD8^+^ T-cell dysfunction, and blocking PD-L1 effectively inhibited tumor growth and improved the survival of tumor-bearing mice (115). IRE-induced immune responses have only recently been of interest to researchers, and the number of relevant studies is fewer than studies of the therapeutic effect of IRE. The various mechanisms should be a focus of future studies to provide a sufficient theoretical basis for clinical application.

## Therapeutic Effects of IRE Combined With Immunotherapy on Solid Tumors

A clinical study (92) evaluated the immunomodulatory effect of IRE to identify an ideal time point for potential adjuvant immunotherapy. The result suggested that most IRE treatment-mediated Treg attenuation occurred between 3 and 5 days after IRE ablation, which provided a window for potentiating clinical efficacy in combination with immunotherapy. Many preclinical and clinical studies have confirmed the favorable efficacy of combination therapy. **Table 2** summarizes the detailed information on the effect of IRE combined with different immunotherapies on the survival and immune status of tumor-bearing models. Murine orthotopic pancreatic cancer studies explored the efficacy of IRE combined with DC vaccination (116), a PD-1 inhibitor (91), and a PD-1 inhibitor combined with a Toll-like receptor-7 (TLR7) agonist (108). The results showed that the combination therapy significantly prolonged overall survival and improved the immune status, enabling antitumor effects in the tumor-bearing mice. Similarly, four clinical studies also verified the outcome benefits and enhanced immune status induced by IRE combined with immunotherapy agents or cells in advanced pancreatic cancer patients (23, 117–119). In addition, some animal studies have shown that IRE combined with immune checkpoint inhibitors (ICIs) and immunostimulants can augment immune status and inhibit the growth of tumors transplanted in the liver (120, 121), skin (121), and prostate (21), yielding extended survival benefits. Two clinical studies found that the IRE and allogenic natural killer cell immunotherapy combination is a promising strategy to enhance antitumor efficacy in advanced hepatocellular carcinoma patients (122, 123). These encouraging results will prompt the development of a combination therapeutic strategy for the treatment of cancer patients refractory to other therapies.

**Table 2:** The information of the effect of IRE combined with different immunotherapies on the survival and immune status of tumor-bearing body.

Species and tumor model	Tumor stage	Number of cases	Combined immunotherapy	Follow-up indicators	Follow-up time	Impact on immune status
Murine orthotopic PDAC	NA	56	Dendritic cell vaccine	Median OS; OS	90 days	Increased the infiltration of CD8^+^ T cells and granzyme B^+^ cells in tumor (116)
Murine orthotopic PDAC	NA	NA	PD-1 inhibitor	Median OS; OS	60 days	Promoted selective infiltration and proliferation of CD8^+^ T cells, with a long-term memory immune response (91)
Murine orthotopic pancreatic cancer	NA	NA	PD-1 inhibitor and Toll-like receptor-7 stimulation	OS	6 months	Increased the infiltration of CD45^+^ cells and CD8^+^ T, DCs, and CD8+IFN-r^+^ T cells in tumor tissue (108)
Human LAPC	Stage III	10	PD-1 inhibitor (nivolumab)	Median OS; OS	Until the date of death	Decreased the circulating Tregs and induced the expression of PD-L1 *in vitro* (117)
Human pancreatic cancer	Stage III/IV	67	Allogeneic NK cell	Median PFS; median OS	Until the date of death	Increased the density of CD4^+^ T, CD8^+^ T, NK, and B cells and the Th1 cytokine levels (118)
Human LAPC	Stage III/IV	62	Allogenic Vγ9Vδ2 T cells	Median PFS; median OS	Until the date of death	Increased the level of IL-2, IFN-γ, TNF-β and NKG2D, αβT, NK cells, and CD44^+^ cells (23)
Human LAPC	Stage III	92	Allogeneic NK cells	OS; DFS	29 months	Increased the density of lymphocyte and enhanced their function, as well as the levels of serum IL-2, TNF-β, and IFN-γ (119)
Murine HCC	NA	60	PD-1 inhibitor	Tumor volume	21 days	Increased the density of CD8^+^ T cells and decreased Tregs in both peripheral blood and tumor tissue (120)
Murine models of melanoma and HCC	NA	40-64	Intratumoral STING agonist	Tumor size	Until the mean tumor diameter was greater than 20 mm^2^	Increased the density of IFN-γ/TNF-α-producing CD4^+^ T and CD8^+^ T cells and delayed tumor growth (121)
Murine prostate carcinoma	NA	14	CTLA-4 inhibitor PD-1 inhibitor	OS	53 days	Promoted robust expansion of tumor- specific CD8^+^ T and memory T cells in blood, tumor, and non-lymphoid tissues (21)
Human primary liver cancer	Stage III/IV	40	Allogenic NK cell	PFS; OS	Until the date of death	Shifted the balance of Th1/Th2 and activated cellular immunity (122)
Human HCC	Stage IV	40	Allogenic NK cell	Median OS; OS	Until the date of death	Augmented the immune functions of the patients (123)

CTLA-4, cytotoxic T lymphocyte antigen 4; DFS, disease-free survival; HCC, hepatocellular carcinoma; LAPC, locally advanced pancreatic cancer; NA: not available; NK, natural killer; OS, overall survival; PFS, progression-free survival; PDAC, pancreatic ductal adenocarcinoma; PD-1, programmed cell death protein-1; STING, stimulator of interferon genes.

## Concluding Remarks

By analyzing the characteristics and related mechanisms of the IRE-mediated immune response, we found that IRE can overcome an immunosuppressive TME, enhance tumor immunogenicity, and activate the cellular and humoral antitumor immune responses of the body, which induces an “*in-situ* vaccination” effect. However, there are still some issues that deserve attention in future studies.

The immune response induced by IRE is significantly different or even contradictory in many preclinical studies. The main reasons for these differences may result from the following reasons. a) The methods for establishing tumor models are different. Subcutaneous and orthotopic xenograft models are mostly used in preclinical studies, while skin and digestive tract tissues originate from the ectoderm and endoderm, respectively. Therefore, their TMEs are significantly different, which leads to different immune responses induced by IRE in the same type of tumor with the same treatment parameters (95, 124). Undoubtedly, animal orthotopic xenograft models are more accurate and suitable for exploring the immune response of tumor-bearing organisms in subsequent studies. b) Different tumor cell lines and mouse strains are used in some studies. In addition, the immune rejection response was not considered in these studies. For example, the mouse hepatoma H22 cell line originates from C3HA mice but is mostly established in C57BL/6 and BALB/c mice to generate hepatoma models. Coincidentally, IRE can always induce significant immune responses in such animal models (86, 120, 124). Therefore, mouse strains and cell-derived mouse strains for animal research must be cautiously selected. c) Several studies have explored the immune response of tumor-bearing models after IRE combined with other therapies (91, 108), while the effect of other therapies on the immune response remains to be further refined and distinguished. In addition, the tumor tissue sampling site, the detection time after IRE treatment, the detection method used, and the immune markers selected to evaluate the immune status can lead to different results. Therefore, investigators should comprehensively consider the relevant influencing factors to ensure the reliability and accuracy of the study results.

Some clinical studies have shown that IRE combined with immunotherapy is effective in prolonging OS in cancer patients (21, 23, 108, 118). However, there is still a lack of large-scale clinical data to support the clinical application of IRE. Future preclinical studies still need to deeply explore the related mechanisms of IRE to determine the optimal strategy of IRE combined with immunotherapy. In addition, for the application of IRE combined immunotherapy, clinical studies need to focus on the selection of patients and explore the best strategy (including timing and dosage) for combining the two approaches.

In summary, with the increasing number of ongoing animal and clinical studies on IRE and its combination with immunotherapy, as well as the development and optimization of IRE instruments, IRE combined with immunotherapy possesses great potential to become a promising choice for patients with unresectable tumors that can benefit more cancer patients.

## Author Contributions

NZ and ZQL retrieved, sorted, and summarized all the related literature and drafted the manuscript. XH and ZZ reviewed and edited the manuscript. YZ and ZhujL helped trace the related paper and edited the manuscript. ZhijL and YL conceived the topic and revised the manuscript. All authors contributed to the article and approved the submitted version.

## Funding

This work was supported by the National Natural Science Foundation of China under Grant No. 81727802.

## Conflict of Interest

The authors declare that the research was conducted in the absence of any commercial or financial relationships that could be construed as a potential conflict of interest.

## Publisher’s Note

All claims expressed in this article are solely those of the authors and do not necessarily represent those of their affiliated organizations, or those of the publisher, the editors and the reviewers. Any product that may be evaluated in this article, or claim that may be made by its manufacturer, is not guaranteed or endorsed by the publisher.
