# Supplementary material for: Irreversible Electroporation: An Emerging Immunomodulatory Therapy on Solid Tumors
Source: Front Immunol. 2022 Jan 7;12:811726. doi: 10.3389/fimmu.2021.811726 (PMC8777104; doi:10.3389/fimmu.2021.811726)
Supplement: Supplementary file 1 [file Table_1.docx]

Supplementary table 1 The detailed information of the clinical studies on the effect of IRE on different types of tumor

| Country | Cancer types and numbers of case | Tumor stage | Effectiveness evaluation methods | Effecti-ve rate | Follow-up indicator | Longest follow-up time | | Side effects | Incidence of side effects | Recurrence rate |
| --- | --- | --- | --- | --- | --- | --- | --- | --- | --- | --- |
| India | HCC(n=21) | NA | CT | 100% | PFS | 30 m | | NA | 0 | 24% (49) |
| Japan | HCC(n=5) | NA | CT&EOB-MRI | 83 % | Local recurrence | 310 d | | NA | 0 | 0 (53) |
| German | Primary(n=8)and secondary(n=35)  liver tumor | NA | EC-MRI | 90% | Tumor volume | 48 m | | Moderate cholestasis | 22% | 33% (52) |
| USA | Primary(n=35) and  secondary(n=36)  liver tumors | NA | MRI | 92.2% | OS | 35.7 m | | Liver abscess;  Myocardial infarction | 7.0% | 31.7% (51) |
| USA | Hepatocellular Carcinoma(n=58) | NA | MRI | 92% | PFS | 26 m | | Asymptomatic;  Gastric ulceration; | 19% | 20.7% (7) |
| NA | HCC(n=3)  Hepatic metastasis(n=11) | NA | CT | 86 % | OS | 594 d | | Intraperitoneal bleeding | 29 % | 14.2 % (51) |
| Netherlands | CRLMs(n=51) | Stage IV | CE-MRI | 74% | LTP-free survival | 53 m | | Septicemia;  Bleeding | 40% | 26% (61) |
| Sweden | CRLM(n = 23); HCC(n=8) ;Other hepatic metastases (n=7) | NA | CE-CT&CE-US | 78.9% | Tumors size | 48 m | | Bile duct dilatation;  Stricture of the portal vein and bile duct | 3.3% | 21.1%(3m); 34.2% (6m) (50) |
| Italy | Malignant hepatic  and abdominal tumor (n=16) | NA | CE-US;  CE-CT&MRI | 93.7% | With or without residual tumor | 18 m | | NA | 0 | 0 (48) |
| China | Hilarcholangiocarcinoma (n= 9) | Stage IV | CT& MRI | 88.9% | PFS and OS | 39 m | | NA | 0 | 44.4% (63) |
| USA | LAPC (n=50) | Stage IV | NA | NA | The median OS time | 18 m | | Upper gastrointestina bleeding; Visceral ulcerations/perforations; Portal vein thromboses. | 17 % | 58% (65) |
| Italy | LAPC (n=10) | Stage IV | CT | 100% | Tumor size;  OS | 24 m | | Pancreatic abscess; Internal fistula in duodenum | 10% | NA (66) |
| Japan | LAPC (n=8) | Stage IV | CE-CT | 100% | Local progression;  OS | 27 m | | Infection; Pseudoaneurysm rupture; Abdominal pain;  Duodenal edema; Nausea;Thrombus | 37.5% | NA (56) |
| Danish | LAPC (n=33) | Stage IV | NA | NA | OS | 53 m | | Pancreatitis; Bleeding duodenal ulcer; Abscess formation; Pancreatic; Cyst formation; | 20% | NA (68) |
| Netherlands | LAPC (n=25) | Stage IV | CT | NA | Local progression; OS and DFS | 16 m | | Edematous pancreatitis; Massive hematemesis; Gastrointestinal complications | 40% | NA (67) |
| Netherlands | LAPC (n=50) | Stage IV | CT | 82% | OS; Tumor size | 72 m | | Abdominal pain ; Pancreatitis; Sepsis ; Gastric leak | 20% | NA (69) |
| Czech | LAPC (n=21) | Stage III | NA | NA | OS; Tumor size | 600 d | | Peripancreatic edema;  Pancreatic or peripancreatic necrosis | 23.8% | NA (57) |
| China | LAPC (n=25) | Stage IV | CT&MRI; Serum-CA199 | 100% | VAS and KPS | 90 d | | Pancreatic fistulas; Gastric emptying; Acute pancreatitis;  Upper gastrointestinal hemorrhage;  Portal vein thrombosis. | 36% | NA (70) |
| Poland | LAPC(n=2);  CRLM(n=1) | Stage IV | IOUS;  Serum-CA199 | 100% | Scar size | 5 m | | 0 | 0 | 0 (62) |
| USA | LAPC(n=200) | Radiographic stage III | CT&MRI | 98% | PFS and OS | 51 m | | Upper gastrointestinal bleed | 37% | 3% (38) |
| Korea | Pancreatic cancer(n=12) | Stage IV | CE-CT | 100% | Median OS and PFS | 15 m | | Abdominal pain;  Acute pancreatitis | 75% | NA (64) |
| Sweden | LAPC(n=5) | NA | CE-US | 100% | Recurrence | 6 m | | NA | 0 | NA (72) |
| German | RCC(n=7) | N/A | MRI | 57.1% | With or without residual tumor | 36 m | | NA | 0 | NA (75) |
| China | RCC (n=15) | N/A | CE-CT | 93.3% | Local recurrence;  Distant recurrence | 34 m | | NA | 0 | 0 (74) |
| German&  France | Lung cancer(n=23) | NA | PET-CT | 39% | local control rate | | 24 m | Pneumothorax | 48 % | NA (59) |
| China | Metastaticretroperitoneal tumors(n=3) | NA | CEUS | NA | Tumor size & OS | | 11 m | NA | NA | NA (78) |
| USA | Pancreatic cancers(n=84)  Liver lesions(n=17)  lung, kidney, mediastinal, pelvic and prostate cancers(n=6) | Stage IV | CT& MRI | 95.3% | LRFS | | 26 m | Bleeding;  Biliary complications;  DVT/PE | 17.9% | 5.9% (73) |
| German | Prostate cancer(n=429) | NA | MRI | 100% | Local control;  LRFS | | 6 years | Urinary continence;  Erectile function | 5.1% | 10% (76) |
| USA | Colorectal cancer liver metastases (n=21); HCC (n=2); Metastatic pancreatic neuroendocrine tumor (n=2); Ampullary carcinoma metastasis (n=1); Hemangiopericytoma (n=1); Leiomyosarcoma metastasis (n=1) | NA | CT & MRI | 100% | Ablation zone size | 6 m | | NA | NA | 5.6% (55) |
| USA | Colorectal metastasis(n=20), Hepatocellular carcinom (n=14); Non-small cell lung cancer (n=2);  Breast cancer(n=2); RCC(n=1);  Carcinoid/neuroendocrine tumors(n=3); Melanoma(n=1);  Soft tissue tumor(n=1) | NA | CT | NA | LRFS | 2 years | | Dehydration;  Biliary stent occlusion;  Cholangitis;  Acute renal failure | 10% | 9.0% (54) |

* CRLM: colorectal liver metastases; CT: computed tomography; CE-CT: contrast-enhanced computed tomography; CE-US: contrast-enhanced ultrasound; DVT: Deep vein thrombosis; EOB-MRI: gadoxetic acid-enhanced MRI; IOUS: intra-operative ultrasound; KPS: karnofsky performance score; LAPC: locally Advanced Pancreatic Cancer; LRFS: local recurrence free survival; LTP-free survival: local tumor progression free survival; MRI: Magnetic resonance imaging; NA: not applicable; OS: overall survival; PE: pulmonary embolism; PFS: progression-free survival; RCC: small renal cell carcinoma; VAS: visual analog scale
